# A voyage from the ER: spatiotemporal insights into polarized protein secretion in neurons

**DOI:** 10.3389/fcell.2023.1333738

**Published:** 2023-12-22

**Authors:** Noortje Kersten, Ginny G. Farías

**Affiliations:** Cell Biology, Neurobiology and Biophysics, Department of Biology, Faculty of Science, Utrecht University, Utrecht, Netherlands

**Keywords:** neurons, polarized protein sorting, endoplasmic reticulum, ERES, Golgi apparatus, conventional protein secretion, unconventional protein secretion

## Abstract

To function properly, neurons must maintain a proteome that differs in their somatodendritic and axonal domain. This requires the polarized sorting of newly synthesized secretory and transmembrane proteins into different vesicle populations as they traverse the secretory pathway. Although the trans-Golgi-network is generally considered to be the main sorting hub, this sorting process may already begin at the ER and continue through the Golgi cisternae. At each step in the sorting process, specificity is conferred by adaptors, GTPases, tethers, and SNAREs. Besides this, local synthesis and unconventional protein secretion may contribute to the polarized proteome to enable rapid responses to stimuli. For some transmembrane proteins, some of the steps in the sorting process are well-studied. These will be highlighted here. The universal rules that govern polarized protein sorting remain unresolved, therefore we emphasize the need to approach this problem in an unbiased, top-down manner. Unraveling these rules will contribute to our understanding of neuronal development and function in health and disease.

## Introduction

Neurons are highly compartmentalized cells with morphologically distinct axonal and somatodendritic domains with specialized functions. Essential transmembrane and secretory proteins are distributed in a polarized manner to these distinct domains. For many years we have assumed that all transmembrane/secretory proteins (hereinafter called cargoes) follow the same rules described in unpolarized cells, in which these proteins are synthesized in the ribosome-harboring rough endoplasmic reticulum (ER) and sorted to the nearby Golgi Apparatus (GA), both primarily localized in the soma of neurons. Thus, it is largely accepted that most cargoes are centrally synthesized in the cell soma and then sorted and transported to the axon and dendrites. This makes the task of maintaining a polarized cargo distribution particularly challenging.

The correct sorting of proteins from the ER to the plasma membrane (PM) is organized in multiple steps in which they are concentrated and packaged into different vesicle populations. The cytoskeletal network and the motors that move cargoes and organelles across it have been reviewed (Aiken and Holzbaur, 2021; Koppers and Farías, 2021; Iwanski and Kapitein, 2023). Here, we will focus on the sorting of cargoes within the secretory pathway and the transfer of proteins between membranous organelles. Cargo exit from the GA has been for a long time considered to be the main regulator of neuronal polarity, ensuring the packaging of cargo into the right vesicle for transport to their destination. However, new evidence suggests that polarized trafficking is initiated early on, from the ER. In addition, examples of sorting of newly synthesized cargoes independent of the centralized GA have been described, adding another layer of complexity for polarized cargo secretion in neurons.

Here, we discuss these recent advances in cargo secretion, offering an integrated view of protein secretion in neurons. Many questions remain open, highlighting the need for an unbiased, top-down characterization of how different cargoes navigate the secretory pathway to reach their destination in polarized neurons. Their morphological complexity makes neurons specifically susceptible to trafficking defects, leading to neurodevelopmental and neurodegenerative disorders, demonstrating the importance of a more comprehensive characterization of their secretory pathways.

### Exploring the ER exit of secretory cargoes

The secretory pathway starts at the rough ER. Cargoes produced here are then confined to specialized regions of the ER, called ER exit sites (ERES). At the ERES, the membrane is coated with COPII-proteins (Barlowe et al., 1994). One of these proteins, Sec24, recognizes cargoes, directly if the cargo has a cytoplasmic domain, or indirectly through cargo receptors. There are four Sec24 paralogs in mammalian cells and they are subdivided into two families considering their homologies: Sec24A/B and Sec24C/D (Pagano et al., 1999). They can interact with both amino acid motifs and folded epitopes on secretory cargoes (Gomez-Navarro and Miller, 2016). Many cargoes have a differential affinity for one of the two subfamilies. A recent systematic review has compared 45 mammalian studies and summarized paralog-specific cargo proteins and their binding motif (Chatterjee, et al., 2021). Notably, in this review, the affinity to one or more Sec24 paralogs for many neurotransmitter transporters has been described. For instance, the serotonin transporter is specific for Sec24C, while noradrenaline, glycine and GABA transporters have been shown to interact with Sec24D. It is tempting to speculate that different Sec24 paralogs are recruited to different ERES to recognize polarized cargoes. However, there is no evidence of any spatial segregation of the four Sec24 paralogs across different ERES or neuronal compartments. A comprehensive overview of paralog-specificity for neuronal cargoes is lacking and it is not clear how this may contribute to cargo polarity. The sorting from the ER can be even more challenging. Some neuronal receptors need to be pre-assembled as a complex in the ER prior to their exit. For instance, the dendritic glutamate AMPA receptor GluA. Recent evidence has shown that the GluA tetramers coassemble and exit the ER by coordinated action of several ER-resident co-factors, some of them trafficked together with GluA to the PM (Schwenk et al., 2019). It remains largely unknown if the assembly of protein complexes in the ER results in more efficient trafficking, and possibly reduces the risk of mislocalization.

**FIGURE 1:**
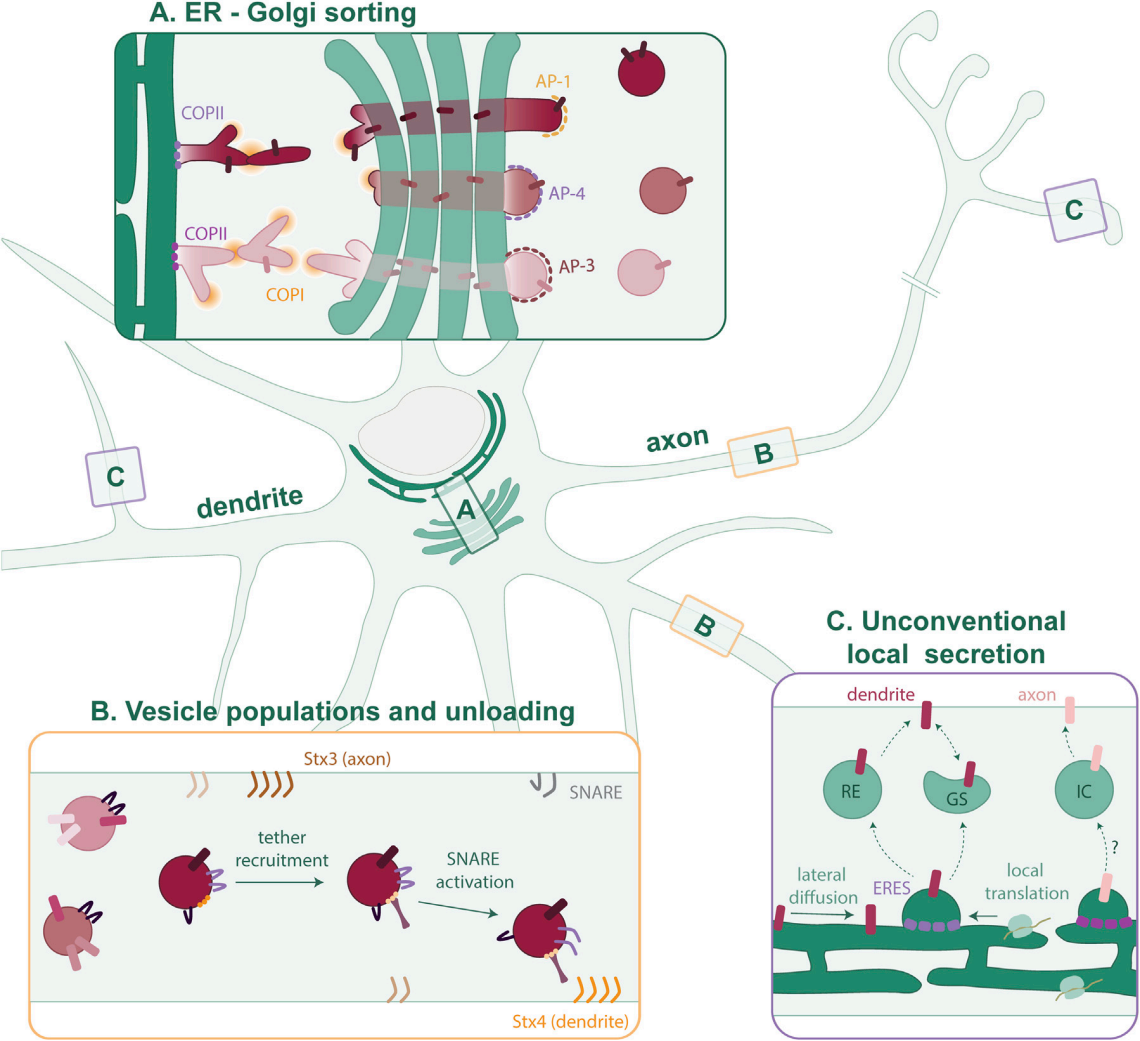
Putative model of cargo sorting in polarized neurons. **(A)** Polarized cargoes are sorted by distinct COPII isoforms at the somatic ERES. This sorting is maintained as the cargo is trafficked through a tubular network via COPI to the cis-Golgi. Segregated cargoes traverse the GA to exit at the TGN in distinct nanocolumns. At the TGN, the different AP complexes concentrate cargoes and mediate vesicle formation. **(B)** Many different vesicle populations exist in the axon and dendrites. It is not clear which cargoes are trafficked together and to what extent the vesicle composition is fluidic. As the vesicles mature, tethers recognize specific GTPases and phosphoinositides on the membrane. SNARE proteins are activated, and the vesicle then fuses with the PM. **(C)** In neurons, proteins can also diffuse laterally within the continuous ER and be confined to peripheral ERES, or they can first be locally synthesized. In dendrites, local cargo secretion involves Recycling Endosomes (REs) and Golgi satellites (GSs). The local secretion pathway in the axon is largely unknown but could involve an unidentified intermediate compartment (IC).

If there is cargo segregation at the ERES for polarized sorting in neurons, it would be expected that this segregation continues after cargo-budding from the ER membrane, and its trafficking to the GA. Studies in non-neuronal cells suggest this possibility. The specificity of Sec24 paralogs towards different SNAREs for vesicle fusion has been shown in cell lines, e.g., the interaction of Sec24C/D with Syntaxin5 (Mancias and Goldberg, 2008) and the interaction of Sec24 A/B with Sec22b (Mancias and Goldberg, 2007; Adolf et al., 2016). Interestingly, new evidence has challenged the view of what happens after COPII coat formation. Initially, it was believed that COPII vesicles bud off and multiple vesicles undergo homotypic fusion to form the ERGIC. From there, COPI vesicles traffic to the cis-Golgi (Scales et al., 1997). More recently, it has been suggested that the ER-ERGIC interface consists of a series of interconnected tubules. In this alternative paradigm, the ERES function as gatekeepers that concentrate cargoes and mediate tubule formation. Then, COPI aids the last trafficking step toward the cis-Golgi (Weigel et al., 2021; Aridor, 2022). In this process, different ARF GTPases could regulate ER to GA trafficking, as a recent pre-print shows that different ARFs occupy distinct domains on the tubular network connecting ER and the GA (Wong-Dilworth et al., 2023a *in pre-print*). This new paradigm emerges from studies in unpolarized cells, so it is unclear to what extent this applies to neuronal cells. This mechanism of ER exit could support polarized cargo selection and segregation at ERES, and their continuous segregation at the receiving membrane (Figure 1A).

### Traversing and exiting the Golgi Apparatus

We could speculate that cargo pre-sorting at the ERES results in nanocolumns spanning from the ER to the TGN (Figure 1A). This might aid the formation of distinct vesicle populations at the TGN. Supporting this idea, recent studies show segregation of proteins along the GA. For instance, APP and the β-secretase BACE1 are spatially segregated from the ER throughout their TGN exit, preventing APP cleavage and formation of toxic Amyloid-β (Fourriere et al., 2022). Other examples of cargoes segregated early in the GA are CD-MPR (lysosomal enzyme receptor) and LAMP (lysosomal membrane protein) (Chen et al., 2017). These proposed nanocolumns could hypothetically fit most of the current models of intra-Golgi trafficking (Mironov and Beznoussenko, 2019).

At the TGN, cargo proteins are packaged in transport vesicles. This is mediated by adaptor protein (AP) complexes, recruited by docking factors such as Arf GTPases and/or phosphoinositides. Then, the APs recognize cargoes and recruit scaffolding proteins to form a coat for vesicle budding (for a comprehensive review see Guardia et al., 2018). Interestingly, there are some studies showing specificity of APs for polarized proteins in neurons. AP-1 is known to sort several somatodendritic cargoes such as transferrin receptor (TfR) and NMDA receptors into vesicle populations that move into the dendrites (Farías et al., 2012; Jain et al., 2015; Guardia et al., 2018). AP-3 is involved in the sorting of axonal proteins into synaptic vesicle precursors and dense core vesicles (Asensio et al., 2013; Li et al., 2016; Jain et al., 2023). AP-4 on the other hand has a less clear-cut role in polarized sorting. It has been proposed to form another somatodendritic vesicle population containing AMPA receptors (Matsuda et al., 2008; Moretto et al., 2023). However, AP-4 has also been implicated in the trafficking of autophagosomal, lysosomal and axonal proteins (Mattera et al., 2017; Pace et al., 2018; Ivankovic et al., 2020; Davies et al., 2022; Majumder et al., 2022). Notably, AP-1 and AP-3 complexes are heterogeneous through the expression of different subunit isoforms (Guardia et al., 2018). However, the functions of these AP variants in neurons remain largely unknown.

Small GTPases orchestrate many steps of vesicular transport. Rab2 has been proposed to orchestrate the biogenesis of presynaptic vesicle precursors, since its depletion results in the accumulation of presynaptic proteins at the TGN (Götz et al., 2021). A study in cell lines showed that ARF GTPases have a differential distribution at the TGN (Wong-Dilworth et al., 2023b). Notably, in this study, some ARFs are not observed at the TGN, while an earlier study implicated these ARFs in the biogenesis of DCVs in neuronal cells (Sadakata et al., 2010). Thus, the role of ARFs in TGN export may be cell-type specific. Moreover, the differential distribution of ARFs at the TGN could imply a role in the polarized sorting of proteins in neurons.

Phosphoinositide conversions at the TGN play a major role in the regulation of secretion, as reviewed by (Posor et al., 2022). The TGN is characterized by phosphatidylinositol-4-phosphate (PtdIns4P) which is synthesized by PtdIns 4-kinases (PI4Ks) and promotes the recruitment of adaptor protein complexes. Notably, early research showed that inhibiting PI4Ks disrupted polarized trafficking (Bisbal et al., 2008). Some APs interact with distinct PI4Ks (Craige et al., 2008; Wieffer et al., 2013), hence it is tempting to speculate that specific phosphoinositide conversions have implications for polarized protein sorting, possibly by priming the membranes of different vesicle populations for recognition by the corresponding trafficking machinery.

### The diversity of post-Golgi vesicle populations

Individual packaging of each cargo protein would be highly inefficient and require an extensive set of distinct cargo adaptors and sorting machinery. Instead, there are only a few APs known, and thus, neurons must package multiple cargoes into the same vesicle (Figure 1B). Which cargoes are being trafficked together has yet remained ambiguous.

In the somatodendritic domain, research has shown that AMPA- and NMDA-type glutamate receptors are trafficked separately, by AP-4 and AP-1 respectively (Matsuda et al., 2008; Farías et al., 2012). It is not known whether cargoes sorted by the same AP are also in the same vesicle. Yet, the controlled delivery of vesicles containing the somatodendritic TfR to the axon results in missorting of NMDA receptors, suggesting these cargoes share the same AP1-derived vesicle (Farías et al., 2015).

Early research identified two vesicle populations for protein delivery into the axon: the Piccolo-Bassoon Transport Vesicles delivering active zone (AZ) proteins and the Synaptic vesicle protein Transport Vesicles (Zhai et al., 2001; Cai et al., 2007). Since then, the delivery of these two populations separately or together into the axon has been unclear. Moreover, it has been suggested that a large fraction of endo-lysosomal cargo clients can be transported within axonal carriers containing SV and AZ proteins, as demonstrated recently across various cell types and species (Vukoja et al., 2018; Rizalar et al., 2023). Contrarily, another study in rat and mouse hippocampal neurons showed that only a minor fraction of SVs and lysosomal proteins are trafficked together (De Pace et al., 2020). Even if there is a small population of hybrid compartments, it would be important to determine its nature, function and regulation. Overall, we lack comprehensive knowledge of different vesicular carriers, their cargoes, and the machinery involved in polarized sorting. It remains largely unexplored to which extent cargoes with distinct destinations or functions are trafficked in the same or distinct vesicles. It cannot be excluded that cargoes can traverse the cell via multiple transport pathways and further investigations might give insights into how the relative contributions of these pathways shift during development, in response to stimuli and across neuron-specific cell types.

### Cargo transfer and unloading

Secretory trafficking is concluded with fusion of the vesicle with the PM (Figure 1B). Membrane targeting and fusion are mediated by many regulatory proteins, but SNARE proteins perform the final fusion step. The mechanism of SNARE fusion has been recently reviewed (Jahn et al., 2023). Tethers are recruited to the membrane by small GTPases and phosphoinositides, which together have been described to establish membrane identity (Behnia and Munro, 2005; Koike and Jahn, 2019). SNAREs can coordinate fusion sites by forming nanoclusters on lipid microdomains in the PM (Chamberlain et al., 2001; Lang et al., 2001). In neurons, Syntaxin 4 (Stx4) localizes to the somatodendritic domain and Syntaxin 3 (Stx3) localizes to the axon. Interfering with Stx3 results in the mislocalization of axonal membrane proteins (Soo Hoo et al., 2016). Yet, many other SNAREs have been shown to be required for axonal outgrowth and SNARE complexes in the axon do not always contain Stx3 (Winkle et al., 2014; Grassi et al., 2015; Fuschini et al., 2018; Wojnacki et al., 2020). Which subset is required could depend on the developmental stage or cell type. In the somatodendritic domain, Stx4 forms exocytic zones next to the postsynaptic density for the exocytosis of NMDA-type glutamate receptors (Kennedy et al., 2010). However, other SNARE complexes, not necessarily containing Stx4, mediate exocytosis of TfR, AMPA and GABA_A_ receptors (Gu et al., 2016; Bakr et al., 2021). Notably, SNAREs can also be present on vesicles as cargo, while not being involved in vesicle targeting or fusion. Which SNAREs on a vesicle are activated is highly regulated. The combinatorial action of tethers, SNAREs, phosphoinositides, GTPases and Sec1/Munc18-like (SM) proteins contributes to the specificity of vesicle fusion (Koike and Jahn, 2022). Further research is necessary to investigate how SNAREs are regulated in neurons and how this contributes to the specificity of vesicle fusion.

### Local translation and secretion: unconventional pathways in the axon and dendrites

The distribution of the main secretory organelles (rough ER and GA) in the soma, and the neuron-specific need to rapidly change their proteome locally in response to stimuli are two seemingly irreconcilable statements. Yet, neurons are more complex than unpolarized cell types that have formed the foundation for our understanding of the secretory pathway. Mounting evidence suggests that (transmembrane) proteins are also locally synthesized in dendrites and axons (Figure 1C) (Cajigas et al., 2012; Holt et al., 2019).

Early research demonstrated the presence and function of ERES complexes in the dendrites (Horton and Ehlers, 2003; Aridor et al., 2004). Locally secreted cargoes can either traffic to the PM directly via an unconventional pathway or progress through Golgi outposts or satellites present in the dendrite (Mikhaylova et al., 2016; Kemal et al., 2022; Wang et al., 2023). The lack of complete Golgi machinery poses the possibility that unconventional secretion pathways could be an important mechanism of delivery. This has been described for a glutamate receptor, which can reach the dendritic surface independently from the Golgi via recycling endosomes (Bowen et al., 2017). This mechanism could extend to many more neuronal membrane proteins, as work by Hanus et al., (2016) demonstrated the presence of various core glycosylated (i.e., not processed by Golgi enzymes) proteins on the neuronal surface. The glycosylation status of proteins such as ion channels affects their properties, thus, core-glycosylation modulates synaptic signaling. Moreover, upon neuronal activation, Golgi satellites with glycosylation machinery can form and these can process either newly synthesized, locally secreted proteins from the ER or core-glycosylated proteins that are recycled from the surface (Govind et al., 2021). Interestingly, synaptic activity spatially confines the secretion of biosynthetic proteins (Hanus et al., 2014).

Local protein synthesis has also been demonstrated in the axon and its presynaptic compartments (Hafner et al., 2019). Yet, while in the dendrite polysomes (a cluster of ribosomes) are prevalent, they seem to be less abundant in the axon. Instead, they are observed as monosomes scattered through the cytoplasm (Biever et al., 2020). Local translation of transmembrane proteins, however, would require the presence of ER-bound ribosomes. Notably, a recent preprint by Koppers et al. (2022) demonstrated translation-dependent interactions between axonal ER tubules and ribosomes, regulated by specific cues, thereby elucidating how secretory proteins can be locally synthesized within the axon. How these proteins successively exit the ER and traffic to the PM remains a topic for further investigation, yet some secretory organelles for local secretion have been proposed (Merianda et al., 2009; Cornejo et al., 2020). Lastly, Golgi-bypassing mechanisms could play a role in the axon, as the presence of core-glycosylated axonal proteins on the membrane (Hanus et al., 2016) suggests that this may be indeed the case.

## Concluding remarks and future perspectives

In recent years, many advances have been made towards understanding the secretory pathway. Nevertheless, many of these recent studies have been performed in unpolarized cell lines. The extremely polarized architecture of neurons and their need to quickly adapt their proteome in response to stimuli puts exceptional demands on their secretory machinery, making it a highly complex system. Findings in unpolarized cells can thus not be easily extrapolated to neurons. For example, the sorting of APP in neurons differs significantly from other cell types, even from polarized MDCK cells. In MDCK cells, APP distribution is polarized towards the basolateral domain, whereas APP in neurons distributes to the somatodendritic domain (analogous to the basolateral domain in MDCK cells) as well as the axonal domain (Haass et al., 1994; 2012). Moreover, recycling endosomes play an important role in APP trafficking in neuronal cells, but not in unpolarized cells (Tan and Gleeson, 2019). In addition, most research focuses on either the dendrite or the axon, and often, only one or a few cargo proteins are studied at a time. A select group of cargoes receives a lot of attention (glutamate receptors, SV proteins), while others remain largely overlooked. From the study of a single cargo protein, we cannot infer any general sorting rules. It is not clear which classes of cargo are trafficked together and to which extent they do this. Lastly, protein transport involves a cascade of many different interactors, rendering the different compartment compositions fluidic, yet this temporal component does not receive a lot of attention. In all, the current knowledge on neuronal protein sorting is severely fragmented. Notably, due to space limitations, we refrained from discussing the crosstalk between the secretory and endocytic pathways for polarized protein distribution in neurons. The highly debated topic of transcytosis, where proteins are first inserted on the atypical plasma membrane domain and are then endocytosed and transported to the correct domain, adds even more complexity to the process of neuronal protein sorting. Moreover, overexpression of cargo can lead to spill-over into the atypical domain, possibly obscuring the results. Therefore, it is not clear at this moment for which proteins and to what extent the transcytosis route plays a role (Wisco et al., 2003; Yamashita et al., 2017; Ribeiro et al., 2019; Nabb and Bentley, 2022; Watson et al., 2023).

The Retention Using Selective Hooks (RUSH) (Boncompain et al., 2012) and similar systems (Bourke et al., 2021) have greatly advanced our knowledge of the secretory pathway. Now there is an urgent need to develop and apply novel technologies to visualize endogenous protein sorting from the ER, without retaining/releasing cargo. Crispr-editing of neurons has been achieved, which can be used to tag proteins at their endogenous levels although efficiency remains a limitation at least in cultured rat neurons (Gao et al., 2019; Willems et al., 2020). The use of multiple-step labeling, e.g., with Halo-tag, could help in dissecting protein sorting within the secretory and endocytic pathways (Los et al., 2008). More importantly, there is the need for development of novel technologies to dissect the secretory pathway in a more comprehensive way. Recent advances in proximity labeling technologies and spatiotemporal proteomics (Lam et al., 2015; Qin et al., 2023) would allow to elucidate compartment-specific composition over time as cargoes traverse the secretory pathway in neuronal cells. Importantly, the downstream analysis should make use of the increasing amount of (open-access) databases and incorporate (AI-driven) tools for protein-protein interaction or motif prediction. In the coming years, these technological advances will allow us to reveal the fluidic compartments and key players involved in polarized distribution of cargoes in neurons.

## Data Availability

The original contributions presented in the study are included in the article/Supplementary material, further inquiries can be directed to the corresponding author.
